# The emerging roles of microRNAs in pneumoconiosis: pathogenic mechanisms and clinical implications

**DOI:** 10.3389/fcell.2026.1811727

**Published:** 2026-05-13

**Authors:** Xuanyan He, Lanxi Ye, Guangchao Zhou, Xia Li

**Affiliations:** Department of Respiratory and Critical Care Medicine, West China Hospital, Sichuan University/West China School of Nursing, Sichuan University, Chengdu, Sichuan, China

**Keywords:** biomarkers, cytokines, miRNA, pneumoconiosis, signaling pathway

## Abstract

Pneumoconiosis is currently one of the most common occupational diseases in clinical practice. Its pathogenesis has not yet been fully elucidated, and effective clinical treatments are lacking. MicroRNAs (miRNAs) are a class of non-coding single-stranded RNA molecules that regulate gene expression at the post-transcriptional level by binding to target genes, thereby inhibiting mRNA translation or promoting mRNA degradation. Existing studies have shown that miRNAs are closely associated with the development and progression of pneumoconiosis, making them important candidate biomarkers for early screening and diagnosis of the disease. This review summarizes the current research status and advances regarding miRNAs in pneumoconiosis, aiming to provide new insights and approaches for understanding its pathogenesis, as well as for early warning, diagnosis, and treatment.

## 1 Introduction

**FIGURE 1:**
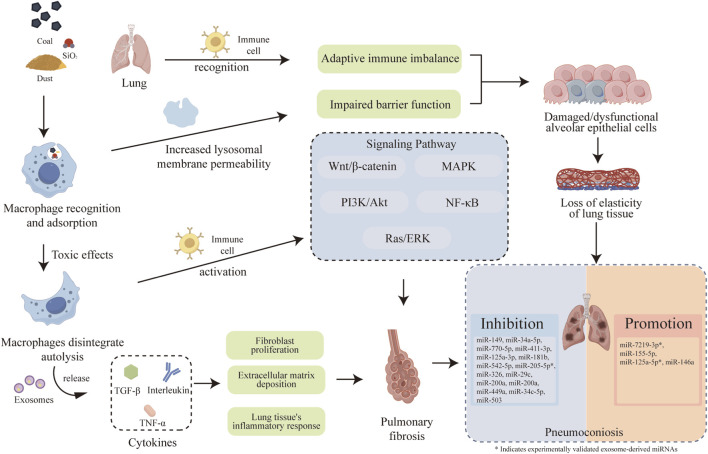
Molecular Mechanisms of miRNA-Mediated Pathogenesis in Pneumoconiosis. The schematic illustrates the complex intracellular signaling pathways and intercellular crosstalk involved in the disease progression. To distinguish regulatory modes, experimentally validated exosome-derived miRNAs (e.g., miR-125a-5p, miR-7219-3p, and miR-205-5p) are specifically denoted with an asterisk (*) to differentiate them from intracellular miRNAs. Key cellular compartments, including alveolar macrophages and alveolar epithelial cells, are explicitly labeled.

Pneumoconiosis is primarily caused by long-term inhalation of hazardous occupational dusts (Krefft et al., 2020). Despite modern industrial hygiene practices, the global burden remains substantial, with an estimated tens of millions of workers facing daily occupational exposure to hazardous particulates worldwide. Recent prevalence trends indicate a concerning resurgence of the disease, particularly severe and accelerated forms of silicosis emerging among younger workers in novel industries such as artificial stone fabrication (Hua et al., 2022). When compared to the disease burden of other fibrotic lung diseases like idiopathic pulmonary fibrosis (IPF)—which predominantly affects older populations—pneumoconiosis is characterized by an earlier onset and a significantly higher loss of disability-adjusted life years (DALYs) within the working-age demographic (Li et al., 2020; Richeldi et al., 2017). According to recent epidemiological data, pneumoconiosis accounts for a significant proportion of occupational respiratory diseases worldwide, with silicosis and coal workers' pneumoconiosis (CWP) driving the highest morbidity and mortality rates, often progressing to irreversible fibrosis even after exposure ceases (Momtazmanesh et al., 2023; Burki, 2021). These dust particles enter the lungs through the mouth and respiratory tract, leading to fibrosis of lung tissue. The formation of collagen fiber nodules results in the loss of lung tissue elasticity and increased hardness, causing ventilation disorders and ultimately leading to pneumoconiosis. Simultaneously, the toxic effects of dust induce the disintegration and autolysis of macrophages, releasing a large number of chemokines, cytokines, and inflammatory mediators. This triggers inflammatory responses in lung tissue, proliferation of fibroblasts, and deposition of extracellular matrix, thereby promoting the occurrence and progression of pulmonary fibrosis (Adamcakova and Mokra, 2021; Bo et al., 2022; Qin et al., 2021) (Figure 1). As a diverse category of occupational diseases, pneumoconiosis encompasses up to 12 types, with silicosis and CWP being the most prevalent (Vos et al., 2020). Although different subtypes of pneumoconiosis share common end-stage fibrotic features, their specific upstream triggering mechanisms, target cells, and corresponding miRNA expression profiles can vary significantly depending on the nature of the inhaled particles. Pneumoconiosis is prevalent worldwide. However, due to its unclear pathogenesis and lack of effective treatment methods, it has become one of the major global public health issues (Jp et al., 2017; Hall et al., 2019).

MicroRNAs (miRNAs) are a class of non-coding RNAs approximately 19–24 nucleotides in length, which primarily regulate the expression of target genes at the post-transcriptional level by binding to the 3′-UTR region of target mRNAs (Bartel, 2004; Saliminejad et al., 2019). Studies have shown that miRNAs are involved in regulating various physiological functions, including individual development, cell proliferation, apoptosis, and other processes. Moreover, changes in the content and types of miRNAs are highly sensitive, holding significant potential value in disease diagnosis and treatment (Bhaskaran and Mohan, 2014; Zeng et al., 2017). In recent years, emerging evidence has highlighted the critical regulatory functions of specific miRNAs in driving the fibrotic pathogenesis of pneumoconiosis (Wang et al., 2010; Cui et al., 2021). Because their expression profiles shift dynamically in response to toxic dust exposure, these epigenetic regulators hold tremendous potential as non-invasive biomarkers for early detection and disease monitoring. This review summarizes the research progress on miRNAs in pneumoconiosis (Table 1), aiming to provide new insights into the pathogenesis of pneumoconiosis and to offer novel approaches for its early warning, diagnosis, and treatment. To ensure the scientific rigor of our synthesis, the inclusion criteria for the studies summarized in Table 1 required that they: (1) specifically investigated functional miRNAs in confirmed pneumoconiosis clinical samples or established *in vivo*/*in vitro* dust exposure models; (2) clearly identified downstream target genes or signaling pathways; and (3) provided robust experimental validation (e.g., dual-luciferase reporter assays, Western blotting, or qRT-PCR) rather than relying exclusively on bioinformatic predictions.

**TABLE 1:** Characteristics of functional miRNAs and their target pathways in pneumoconiosis.

miRNA	Pneumoconiosis subtype	Species and experimental model	Sample type/Study type	Expression	Direct target and pathway	Validation method	Clinical relevance/effect	References
miR-146a	Silicosis	Human; rat (NR8383 macrophages)	BALF; cells/clinical and *in vitro*	Upregulated	IL-1β (NF-κB)	qRT-PCR, ELISA	Promotes inflammation; Potential biomarker for disease severity	Zhang, et al. (2016b)
miR-149	Silicosis	Mouse; human (A549, HBE cells)	Lung tissue; cells/*in vivo* and *in vitro*	Downregulated	IL-6 (Unstated)	qRT-PCR, Western blot, IHC, ELISA	Negatively regulates IL-6; Delays silicosis progression	Fan et al. (2014)
miR-34a-5p	Silicosis	Mouse; human (A549 cells)	Lung tissue; Cells/*in vivo* and *in vitro*	Downregulated	Smad4 (TGF-β1/Smad4)	qRT-PCR, Western blot, dual-luciferase assay	Inhibits EMT and exerts anti-fibrotic effects	Qi et al. (2020)
miR-770-5p	Silicosis	Mouse; human (MRC-5 fibroblasts)	Serum; lung tissue; cells/clinical, *in vivo* and *in vitro*	Downregulated	TGFBR1 (TGF-β)	qRT-PCR, Western blot, dual-luciferase assay	Inhibits fibroblast activation and pulmonary fibrosis	Yuan et al. (2021)
miR-411-3p	Silicosis	Rat; mouse (fibroblasts)	Lung tissue; cells/*in vivo* and *in vitro*	Downregulated	MRTFA (MRTF-A/SRF)	Microarray, qRT-PCR, Western blot	Exerts anti-fibrotic effects by inhibiting MRTF-A	Gao et al. (2020)
miR-125a-3p	Silicosis	Mouse; human (NIH/3T3, MRC-5)	Lung tissue; Cells/*in vivo* and *in vitro*	Downregulated	Fyn/STAT3 (Fyn/STAT3)	qRT-PCR, Western blot, dual-luciferase assay	Inhibits fibroblast activation and attenuates fibrosis	Xu Q. et al. (2019)
miR-181b	Silicosis	Rat (NR8383 macrophages)	Cells/*in vitro*	Downregulated	TNF-α (Unstated)	qRT-PCR, Western blot, ELISA	Regulates inflammatory cytokine secretion	Zhang et al. (2015)
miR-125a-5p	Silicosis	Mouse	Lung tissue; serum exosomes/*in vivo*	Upregulated	TRAF6 (NF-κB)	qRT-PCR, Western blot, dual-luciferase assay	Regulates T cell subsets; Promotes fibrosis	Ding et al. (2023)
miR-155-5p	Silicosis	Mouse	Lung tissue; Cells/*in vivo* and *in vitro*	Upregulated	Mep-1a (Unstated)	qRT-PCR, Western blot	Promotes fibroblast and macrophage activation	Chen et al. (2020)
miR-542-5p	Silicosis	Mouse	Lung tissue; cells/*in vivo* and *in vitro*	Downregulated	Itgα6 (FAK/PI3K/AKT)	qRT-PCR, Western blot, Dual-luciferase assay	Attenuates fibroblast differentiation and proliferation	Yuan et al. (2018)
miR-205-5p	Silicosis, CWP	Mouse	Lung tissue; EVs/*in vivo* and *in vitro*	Downregulated	E2F1 (ATF4/CHOP)	qRT-PCR, Western blot, dual-luciferase assay	Inhibits pulmonary fibrosis; Regulates autophagy	Qian et al., 2021; Zhao et al., 2025
miR-326	Silicosis	Mouse	Lung tissue; Cells/*in vivo* and *in vitro*	Downregulated	TNFSF14, PTBP1 (Unstated)	qRT-PCR, Western blot, dual-luciferase assay	Inhibits inflammation and promotes autophagy	Xu T. et al. (2019)
miR-29c	Silicosis	Mouse	Lung tissue/*in vivo*	Downregulated	Unstated (Wnt/β-catenin)	qRT-PCR, Western blot	Suppresses silica-induced lung fibrosis	Wang et al. (2018a)
miR-200a	Silicosis	Mouse	Cells/*in vitro*	Downregulated	Unstated (Wnt/β-catenin)	qRT-PCR, Western blot	Regulates EMT in lung epithelial cells	Wang et al. (2018b)
miR-449a	Silicosis	Mouse	Lung tissue; cells/*in vivo* and *in vitro*	Downregulated	Bcl2 (MAPK/ERK)	qRT-PCR, Western blot, dual-luciferase assay	Regulates autophagy and inhibits pulmonary fibrosis	Han et al. (2016)
miR-34c-5p	Silicosis	Human (HBE cells)	Cells/*in vitro*	Downregulated	FOSL1 (PTEN/PI3K/Akt)	qRT-PCR, Western blot, dual-luciferase assay	Inhibits EMT, proliferation, and migration	Pang et al. (2022)
miR-503	Silicosis	Mouse	Lung tissue; cells/*in vivo* and *in vitro*	Downregulated	PI3Kp85 (PI3K/Akt)	qRT-PCR, Western blot, dual-luciferase assay	Modulates EMT and pulmonary fibrosis	Yan et al. (2017)
miR-7219-3p	Silicosis	Mouse	Macrophage-derived exosomes/*in vivo* and *in vitro*	Upregulated	SPRY1 (Ras/ERK)	qRT-PCR, Western blot, ultracentrifugation	Promotes fibroblast trans-differentiation (FMT)	Niu et al. (2022)

Unlike previous literature reviews that primarily catalog individual miRNAs, this review provides a uniquely integrated perspective. First, we systematically differentiate the epigenetic networks between distinct pneumoconiosis subtypes and map their specific cellular contexts. Second, we place a strong emphasis on the crucial, yet previously under-discussed, roles of exosome-mediated intercellular communication within the pulmonary microenvironment. Third, this review comprehensively integrates both early diagnostic biomarker evaluation and targeted therapeutic applications, bridging traditional bulk-tissue findings with emerging single-cell transcriptomic insights. Finally, we systematically incorporate the most recently identified functional miRNAs—such as the exosome-derived miR-7219-3p—to provide an updated and holistic view of converging profibrotic signaling hubs.

## 2 The pathogenesis of pneumoconiosis

### 2.1 Direct cytotoxicity

SiO_2_, dust, and coal can initiate free radical reactions, triggering peroxidation of the alveolar macrophage membrane and generating lipid peroxides, which subsequently affect fibroblasts, leading to their functional activation, excessive collagen release, and the formation of diffuse pulmonary collagen fibrosis. The damaging effect of nano-SiO_2_ on the cell membrane exhibits a certain degree of size dependency (Kim et al., 2019). As the size of nanoparticles decreases, the damage to the cell membrane intensifies, resulting in the generation of more free radicals in aqueous media, disruption of the plasma membrane, release of lysosomal enzymes, and ultimately tissue damage (Deville et al., 2020).

### 2.2 Generation of highly reactive molecules

Highly reactive molecules (free radicals), such as reactive oxygen species (ROS) radicals and nitrogen radicals, when produced in excess, can lead to oxidative damage to macromolecules in the body, including nucleic acids, lipids, and proteins. Nitric oxide (NO) plays a significant role in the pathogenesis of silicosis. NO is produced from L-arginine through the action of inducible nitric oxide synthase (iNOS), converting it to L-citrulline. It then interacts with superoxide to form peroxynitrite, which damages mitochondria and DNA, and inactivates various proteins (Castranova et al., 1998; Pacher et al., 2007). The interaction between alveolar macrophages and SiO_2_ particles results in elevated levels of inducible NOS and the generation of ROS, leading to damage to lung cells (Fubini and Hubbard, 2003).

### 2.3 Cytokine production

Oxidative stress activates transcription factors such as nuclear factor-κB (NF-κB) and activator protein 1 (Yang H. et al., 2019). When SiO_2_ interacts with alveolar macrophages and pulmonary epithelial cells, it translocates NF-κB from the cytoplasm to the nucleus, where it binds to DNA and participates in the transcription and translation of genes involved in inflammatory and fibrotic processes. Marrocco et al. (Tang et al., 2022) found that SiO_2_ can induce the overexpression of tumor necrosis factor-α (TNF-α) in the lung tissues of dust-exposed rats during early inflammatory injury, indicating that TNF-α plays an important role in the local damage and inflammatory response stages of silicosis. The release of cytokines, chemokines, lipid mediators, and growth factors leads to the accumulation of polymorphonuclear cells and monocytes in the alveolar spaces and around SiO_2_ particles, promoting inflammatory responses and granuloma formation (Zhao et al., 2020). Importantly, the intensity and duration of this cytokine-driven inflammatory response are not merely passive reactions but are tightly modulated by post-transcriptional regulators. As detailed in the subsequent sections, various miRNAs act as critical rheostats—either amplifying or dampening the expression of key transcription factors (like NF-κB) and inflammatory mediators (like TNF-α and interleukins)—thereby dictating the severity of the initial tissue injury.

### 2.4 Fibrosis

Leukotriene B4 is a pro-inflammatory lipid mediator that facilitates mast cell responses to SiO2 particles. The chemokines monocyte chemoattractant protein-1 and macrophage inflammatory protein-2 are involved in SiO_2_ particle-induced lung injury (Ferreira et al., 2020). Alveolar macrophages are the first to interact with SiO_2_ particles and activate a cascade of extracellular signals. Under the action of arginase, alveolar macrophages polarize toward the M2 phenotype, which promotes inflammation resolution and tissue repair (Lopes-Pacheco et al., 2013). Crucially, this M1/M2 macrophage polarization is highly orchestrated by specific miRNAs (Kishore and Petrek, 2021). Dysregulated miRNAs can skew macrophages toward a pro-fibrotic M2 phenotype, sustaining abnormal tissue repair and driving the irreversible progression of pneumoconiosis (Ge et al., 2024). In alveolar macrophages, class A scavenger receptors and the macrophage receptor with collagenous structure (MARCO) recognize and phagocytose SiO_2_ particles, thereby clearing them from the alveoli (Yang M. et al., 2019). Failure to successfully remove these particles from the lungs can lead to persistent inflammatory responses. Prolonged interactions between immune cell populations—such as neutrophils, macrophages, and epithelial cells—and dust particles may ultimately result in pulmonary interstitial fibrosis. MARCO-mediated mitochondrial apoptosis plays a significant role in the development of silicosis. Studies have shown (Xie et al., 2021; Porter et al., 2002) that early preventive intervention using signal transduction inhibitors in silica-dust-exposed rats yields better outcomes than therapeutic interventions administered after silicosis has already formed. During the inflammatory process induced by particles, the secretion of interleukin-1α (IL-1α) and interleukin-1β (IL-1β) by pulmonary epithelial cells and alveolar macrophages increases (Aboushousha et al., 2021; Bellon et al., 2017).

Furthermore, the increase in IL-1 and TNF-α can stimulate the expression of IL-6, which is also an inflammatory mediator involved in the progression of silicosis (Uciechowski and Dempke, 2020). The increased expression of matrix metalloproteinase-2 (MMP-2) and MMP-9 serves as a key regulatory factor in extracellular matrix degradation during the pathogenesis of silicosis (Xue et al., 2017). The resulting imbalance between extracellular matrix degradation and collagen deposition is a significant contributor to granuloma formation, lung parenchymal injury, and pulmonary fibrosis. Recent advances have shifted the paradigm, revealing that this pathological imbalance between extracellular matrix (ECM) degradation and collagen deposition is actively governed by dysregulated epigenetic networks. miRNAs play pivotal roles at this stage by directly targeting core profibrotic signaling hubs, functioning as essential molecular switches that determine whether activated fibroblasts undergo apoptosis or drive progressive, irreversible pulmonary fibrosis.

### 2.5 Apoptosis

Alveolar macrophage apoptosis is an early pathological event in pulmonary fibrotic injury, playing a significant role in the development and progression of silica dust-related diseases, primarily mediated by mitochondrial dysfunction and increased expression of death receptors and their ligands (Chen et al., 2021). After SiO_2_ particles are phagocytosed by alveolar macrophages, they can upregulate the expression of pro-apoptotic genes, ultimately leading to cell apoptosis. In the process of SiO_2_-induced pulmonary fibrosis, alveolar macrophage apoptosis is considered the pathological basis for the formation of alveolitis, which leads to pulmonary fibrosis. In addition to oxidative stress, SiO_2_ particles can also cause loss of mitochondrial membrane potential, subsequently activating caspase-9 and caspase-3 and inducing DNA fragmentation (Deville et al., 2020). During apoptosis, chemokines are released, attracting new inflammatory cells and amplifying the inflammatory response. Alveolar macrophages undergoing apoptosis also release SiO_2_ particles back into the lung parenchyma, where they are re-phagocytosed by other alveolar macrophages, thereby prolonging the cycle of tissue damage. Crucially, the apoptotic fate of these alveolar macrophages and the subsequent amplification of the inflammatory cascade are intricately programmed by specific apoptosis-related miRNAs. By regulating mitochondrial membrane potential and apoptosis-associated genes, these miRNAs serve as a critical mechanistic bridge connecting initial dust cytotoxicity to long-term fibrotic remodeling.

## 3 Biomarker potential of miRNAs in pneumoconiosis

Given their remarkable stability in biofluids and cell-specific expression patterns, dysregulated miRNAs have emerged as highly promising diagnostic and prognostic tools in clinical occupational medicine (Zhang et al., 2016a). To clarify the role of miRNAs in pneumoconiosis, bioinformatics techniques have been widely employed to analyze the expression levels of miRNAs in the serum of pneumoconiosis patients, investigate their potential target genes, and explore related regulatory networks.

### 3.1 Differential expression profile of miRNAs in patients with pneumoconiosis

By detecting miRNA expression levels and genetic polymorphisms in lung tissue, it can serve as an important means for screening high-risk populations, early diagnosis, and prognosis assessment of pneumoconiosis. Tian et al. (Tian et al., 2023) analyzed the differential miRNA expression profiles in lung tissues from 3 CWP patients, eight silicosis patients, and four healthy controls. They found that, compared with the healthy control group, there were 375 and 88 differentially expressed miRNAs in the lung tissues of CWP and silicosis patients, respectively, while 34 differentially expressed miRNAs were identified between the CWP and silicosis groups. This finding strongly suggests that although CWP and silicosis both belong to pneumoconiosis, their distinct exposure contexts induce partially different epigenetic regulatory networks. Through Gene Ontology (GO) and Kyoto Encyclopedia of Genes and Genomes (KEGG) enrichment analyses, it was revealed that the differentially expressed miRNAs were primarily enriched in signaling pathways such as transforming growth factor-β1 (TGF-β1), mitogen-activated protein kinase (MAPK), and the tumor suppressor gene P53. Zhang et al. (2016a) conducted a differential expression profile analysis of miRNAs in samples from nine different cases of pneumoconiosis. They found that, compared with dust-exposed individuals, stage I pneumoconiosis patients exhibited 123 differentially expressed miRNAs, while stage II patients showed 46. Functional enrichment analysis revealed that these differentially expressed miRNAs play significant roles in various biological functions, including ligand–receptor interactions, inflammation, immunity, and fibrosis.

### 3.2 Study on circulating miRNA as an early diagnostic and prognostic marker for pneumoconiosis

The bloodstream contains abundant and stable circulating miRNAs, which can serve as early non-invasive biomarkers for pneumoconiosis. Zhang et al. (Zhang et al., 2023) analyzed blood samples from 15 pneumoconiosis patients and eight healthy controls, identifying 1,417 differentially expressed mRNAs and 241 differentially expressed miRNAs in the blood of pneumoconiosis patients compared to the healthy control group. However, most miRNA or mRNA expression levels showed no significant differences between early- and late-stage pneumoconiosis patients. Huang et al. (Huang et al., 2018) utilized microarray and bioinformatics techniques to analyze differentially expressed miRNAs in four dust-exposed pneumoconiosis patients and four healthy individuals. They identified 32 upregulated and four downregulated miRNAs, among which has-miR-4516 exhibited the largest fold change. Additionally, through *in silico* target gene prediction (without further *in vitro* experimental validation in this specific study), it was found that hsa-miR-4516 in the serum of pneumoconiosis patients potentially targets genes encoding basonuclin two and inhibitors of growth family member 4. This indicates that has-miR-4516 further regulates the progression of pneumoconiosis by targeting these potential genes. Guo et al. (Guo et al., 2013) conducted an in-depth analysis of serum samples from different stages of CWP and found that the expression levels of certain miRNAs, such as miR-18a, miR-149, and miR-671-3p, were consistently upregulated or downregulated across the three stages of CWP. However, some miRNAs, including the miR-200 family and miR-222 family, exhibited inconsistent expression trends across the three stages. These aberrantly expressed miRNAs demonstrated dynamic expression patterns in the serum samples of CWP patients, suggesting that they may play a significant role in the onset and progression of CWP and hold important implications for the diagnosis and prognosis of pneumoconiosis.

Despite these promising findings, a critical evaluation of the current literature reveals several methodological limitations. Many differential miRNA expression studies are constrained by exceptionally small sample sizes (e.g., cohorts of only 3 CWP patients and eight silicosis patients). This lack of statistical power is compounded by variability between detection platforms (such as RNA-seq *versus* microarray *versus* qPCR), which leads to poor reproducibility across different cohorts. Furthermore, critical confounding factors—including smoking history, co-morbidities, and exact exposure duration—are frequently under-reported. To overcome these hurdles, researchers are exploring novel non-invasive techniques; for instance, recent pilot studies have successfully utilized next-generation sequencing (NGS) for miRNA detection in exhaled breath condensates (Cherchi et al., 2023). Moving forward, establishing true clinical utility will require large-scale, standardized systematic reviews and meta-analyses, as effectively demonstrated in recent evaluations of miRNA biomarkers for asbestos-related lung diseases (Mukhopadhyay et al., 2025).

## 4 Role of miRNAs in pneumoconiosis pathogenesis and regulated signaling pathways

miRNAs play a significant role in various pathophysiological processes of pneumoconiosis. The following discussion will primarily elaborate on their involvement from the perspectives of cytokines, target proteins, and signaling pathways. Importantly, within the complex pulmonary microenvironment, miRNA-mediated regulation is highly cell-type specific. The progression of pneumoconiosis relies on the intricate orchestration of distinct cellular compartments: alveolar macrophages (which initiate the inflammatory cascade upon dust phagocytosis), pulmonary epithelial cells (which undergo damage and epithelial-mesenchymal transition (EMT)), and lung fibroblasts (which act as the ultimate effectors of extracellular matrix deposition). Elucidating the specific cellular origin and intrinsic targets of these miRNAs is essential for decoding the complete fibrotic network. Recently, the advent of single-cell RNA sequencing (scRNA-seq) has revolutionized our understanding of this microenvironment by revealing unprecedented cellular heterogeneity (Adams et al., 2020). scRNA-seq studies in fibrotic lung diseases have identified highly specific pathogenic subpopulations—such as aberrant basaloid epithelial cells, unique pro-inflammatory macrophage states, and highly activated myofibroblast subsets. Integrating miRNA expression profiles with these scRNA-seq-identified subpopulations will be crucial for understanding how specific miRNA targets are dynamically regulated within these distinct cellular niches (Zhou Z. et al., 2024).

### 4.1 miRNA regulates cytokines in silicosis and CWP

#### 4.1.1 Interleukin

When a large amount of occupational dust enters the lung tissue and is engulfed by alveolar macrophages, its toxic effects cause the macrophages to lyse and autolyze, releasing various inflammatory mediators (such as IL-1β, IL-6, and IL-8). This leads to edema, inflammatory infiltration, and leukocyte exudation in the alveoli and lung tissue, subsequently impairing pulmonary function and exacerbating the progression of pneumoconiosis (Dimitrijević et al., 2014). Zhang et al. (2016b) discovered a potential characteristic association between miR-146a and IL-1β in the bronchoalveolar lavage fluid of silicosis patients. By measuring the relative expression levels of miR-146a and IL-1β in patients, it was found that their expression levels peaked at different stages of the disease (stages I, II, and III), respectively. This suggests that IL-1β may induce the upregulation of miR-146a expression, thereby promoting the progression of silicosis. Additionally, the shorter the occupational exposure duration of silicosis patients, the higher the relative expression level of miR-146a, indicating that miR-146a could serve as a key factor for early screening and prevention of silicosis. Transfection of A549 cells with miR-149 agonists and inhibitors revealed that IL-6 expression levels were upregulated following agonist transfection and downregulated after inhibitor transfection. Additionally, in a mouse model treated with SiO_2_ dust, miR-149 expression was found to be downregulated, while IL-6 expression was upregulated. These findings suggest that miR-149 negatively regulates IL-6, thereby delaying the progression of silicosis (Fan et al., 2014).

#### 4.1.2 TGF-β1

TGF-β1 can participate in the EMT process, thereby promoting fibroblast proliferation and extracellular matrix deposition, which leads to a decrease in lung tissue elasticity and pulmonary function, ultimately exacerbating pulmonary fibrosis (Tan and Chen, 2021). At the cellular level, the fibrotic effects of TGF-β1 are orchestrated by distinct miRNA profiles depending on the target cell type. In pulmonary epithelial cells, dysregulated miRNAs often drive EMT, whereas in lung fibroblasts, they predominantly govern the fibroblast-to-myofibroblast transition (FMT) and excessive collagen synthesis (Gregory et al., 2008). The Smad (small mother against decapentaplegic) family serves as characteristic signaling proteins of TGF-β, with different Smads mediating the involvement of the same or different TGF-β family members in signaling pathways. Bioinformatic predictions combined with dual-luciferase reporter assays have experimentally validated that Smad4 is a direct target gene of miR-34a-5p. In a mouse silicosis model, the expression levels of Smad4 and miR-34a-5p were found to be negatively correlated, while the EMT process was also accompanied by a decrease in miR-34a-5p expression. Furthermore, TGF-β1 can induce EMT by suppressing miR-34a-5p expression and increasing Smad4 expression, indicating that miR-34a-5p exerts anti-fibrotic effects through the TGF-β1/SMAD4 signaling pathway (Qi et al., 2020). miR-770-5p and miR-411-3p are involved in the process of silicotic pulmonary fibrosis through the TGF-β1 regulatory mechanism, as evidenced by the downregulation of miR-770-5p expression in the serum of silicosis patients. *In vivo* and *in vitro* silicosis models demonstrate that overexpression of miR-770-5p inhibits the transduction of the TGF-β signaling pathway by targeting transforming growth factor beta receptor 1 (TGFBR1), thereby attenuating SiO_2_-induced pulmonary fibrosis progression. In TGF-β1-induced lung fibroblasts, the expression of miR-411-3p is downregulated. Further analysis reveals that miR-411-3p can effectively inhibit TGF-β1-induced differentiation of lung fibroblasts by targeting myocardin-related transcription factor A (MRTFA), thereby exhibiting antifibrotic effects (Yuan et al., 2021; Gao et al., 2020). In the SiO2-induced silicosis fibrosis model, it was found that the expression level of miR-125a-3p was significantly downregulated and exhibited a negative feedback regulation with TGF-β1. Upregulation of miR-125a-3p expression could attenuate the progression of silicosis fibrosis, while overexpression of Fyn and P-STAT3 could counteract the phenomena induced by miR-125a-3p, indicating that miR-125a-3p exerts anti-fibrotic effects by modulating the Fyn/STAT3 signaling pathway (Xu Q. et al., 2019).

#### 4.1.3 TNF-α

Studies have shown that alveolar macrophages play a significant role in the development of silicosis. As SiO_2_ continuously deposits, macrophages passively release a range of inflammatory mediators, including TNF-α (Yucesoy et al., 2002). Within these macrophages, specific miRNAs function as intrinsic regulators of immune activation and polarization. Zhang et al. (Zhang et al., 2015) transfected rat alveolar macrophages (NR8383 cells) with miR-181b mimics and found that transfection with miR-181b mimics led to a significant decrease in TNF-α levels, whereas knockdown of miR-181b resulted in an upregulation trend in TNF-α expression. Additionally, whether exosomal miRNAs can influence the development of silicosis by regulating T lymphocyte differentiation remains controversial. To address this, Ding et al. (Ding et al., 2023) constructed a miR-125-5p antagonism mouse model of silicosis and discovered that exosomal miR-125a-5p was upregulated in the pulmonary fibrosis of silicotic mice. Furthermore, silencing or knocking out exosomal miR-125a-5p could target and induce tumor necrosis factor receptor-associated factor 6 (TRAF6) to regulate helper T lymphocyte subsets, thereby alleviating the process of silicotic fibrosis.

### 4.2 miRNA regulates target genes/target proteins to influence the progression of silicosis

miRNA regulates the expression of corresponding potential target genes/target proteins, playing a significant role in promoting or inhibiting the process of silicotic pulmonary fibrosis. In an *in vitro* silicosis model, it was found that SiO_2_ can downregulate the expression of Mep-1a (mepria), while upregulation of miR-155-5p inhibits Mep-1a, thereby enhancing the activation of pulmonary fibroblasts and alveolar macrophages and promoting the occurrence of silicotic fibrosis (Chen et al., 2020). Overexpression of miR-542-5p can alleviate the pathological changes of silicotic fibrosis, repair alveolar structural damage, and reduce the formation of silicotic nodules. Additionally, integrin α6 (Itgα6) has been experimentally confirmed as a direct target of miR-542-5p through luciferase reporter assays, and inhibiting this target can reduce the differentiation and proliferation of pulmonary fibroblasts (Yuan et al., 2018). The transcription factor E2F1 has been robustly verified through both bioinformatic screening and molecular assays as a direct target gene of miR-205-5p. Interestingly, the role of miR-205-5p has been validated in distinct subtypes of pneumoconiosis. Upregulation of miR-205-5p expression effectively alleviates the progression of pulmonary fibrosis in silicosis mice, while overexpression of E2F1 can counteract the inhibitory effect of miR-205-5p on pulmonary fibrosis (Qian et al., 2021). Zhao et al. (Zhao, Hao, and Zhang, 2025) found that miR-205-5p derived from pulmonary extracellular vesicles triggers the development associated with CWP via the ATF4/CHOP signaling axis. Through rigorous experimental validation including reporter assays, Zhang et al. (Zhang et al., 2018) found that EZH2 and hnRNPK are the targets of exosomal miR-125a. Overexpression of exosomal miR-125a can inhibit EZH2 and hnRNPK in a targeted manner, thereby impeding the occurrence and progression of silicosis. miRNA can act on multiple target genes/target proteins during the regulation of silicosis. For example, miR-326 inhibits inflammation and promotes autophagy activity by targeting TNFSF14 and PTBP1, thereby alleviating SiO_2_-induced pulmonary fibrosis (Xu T. et al., 2019).

### 4.3 miRNA acts on signaling pathways to influence pneumoconiosis

#### 4.3.1 Wnt/β-catenin signaling pathway

The Wnt/β-catenin signaling pathway is a complex network of protein interactions that plays a crucial role in multiple developmental and disease progression processes. Through a mouse silicosis model and an miR-29c overexpression lentiviral vector, it was found that miR-29c exerts an inhibitory effect on genes associated with the Wnt/β-catenin signaling pathway, indicating that miR-29c suppresses the development of SiO_2_-induced silicotic fibrosis in mice by regulating the Wnt/β-catenin signaling pathway (Wang et al., 2018a). Additionally, miR-200a can also modulate the Wnt/β-catenin signaling pathway to participate in the progression of silicotic fibrosis (Wang et al., 2018b).

#### 4.3.2 MAPK signaling pathway

In silicosis fibrosis, the activation of the MAPK signaling pathway is associated with fibroblast proliferation, differentiation, and extracellular matrix accumulation. Han et al. (Han et al., 2016) found that overexpression of miR-449a in a mouse silicosis model significantly alleviated SiO_2_-induced pulmonary inflammatory lesions and the degree of pulmonary fibrosis. The 3′UTR of Bcl2 was identified as the responsive sequence for miR-449a, and its mechanism of action involves miR-449a mitigating silicosis fibrosis by downregulating Bcl2 expression and reducing autophagy activity in lung fibroblasts. Additionally, *in vitro* experiments demonstrated that TGF-β-induced Bcl2 expression is regulated by the MAPK/extracellular signal-regulated kinase (ERK) pathway.

#### 4.3.3 PI3K/Akt signaling pathway

The PI3K/Akt signaling pathway is regulated by miRNAs and influences the progression of silicotic fibrosis. For instance, Pang et al. (Pang et al., 2022), through bioinformatics analysis and dual-luciferase reporter gene assays, identified Fos-like antigen 1 (FOSL1) as a potential target of miR-34c-5p. Moreover, miR-34c-5p/FOSL1 attenuates SiO_2_-induced proliferation and migration of human bronchial epithelial cells by inhibiting PTEN/PI3K/AKT signaling activity, promotes apoptosis, and thereby prevents the occurrence of EMT. Yan et al. (Yan et al., 2017) found that downregulating the expression of miR-503 alleviates its inhibition of the target gene PI3Kp85, thereby activating the PI3K/Akt signaling pathway and promoting EMT during silica-induced pulmonary fibrosis. Importantly, this process perfectly illustrates the competing endogenous RNA (ceRNA) network and lncRNA-miRNA interactions, as miR-503 is actively ‘sponged' by the long non-coding RNA (lncRNA) MALAT1, adding another layer of upstream epigenetic complexity to the fibrotic cascade.

#### 4.3.4 NF-κB signaling pathway

The NF-κB signaling pathway plays a “switch” role in the inflammatory response during the early stage of pneumoconiosis and the subsequent fibrotic process (Zhou Q. et al., 2024). When dust enters the lungs, macrophages and epithelial cells release a large number of pro-inflammatory factors through this pathway. Recent studies have indicated that miR-125a-5p plays a critical regulatory role in silica-induced pulmonary fibrosis. Ding et al. (Ding et al., 2023) found that silica-stimulated macrophages release exosomes encapsulating miR-125a-5p. Upon uptake by T lymphocytes, these exosomes function by directly targeting TRAF6.

#### 4.3.5 Ras/ERK signaling pathway

The Ras/ERK/MAPK signaling pathway is a central pathway regulating cell growth, differentiation, and fibrosis. In the development of pneumoconiosis, abnormal activation of this pathway is closely associated with the FMT (Xie et al., 2021). Niu et al. (Niu et al., 2022) have identified miR-7219-3p as a key regulator of this pathway, providing a classic example of cross-cellular miRNA regulation. In silicosis models, silica stimulation induces macrophages to release exosomes containing high levels of miR-7219-3p. These exosomes act as intercellular mediators, dynamically delivering miR-7219-3p from the immune compartment directly to lung fibroblasts. Once inside the target cells, miR-7219-3p specifically binds to and suppresses the expression of SPRY1, an endogenous negative feedback regulatory protein of the Ras/ERK pathway. Consequently, miR-7219-3p-mediated downregulation of SPRY1 relieves the inhibition on the pathway, leading to a significant increase in ERK phosphorylation (p-ERK) levels. This sustained activation of the pathway enhances fibroblast proliferation, migration, and the synthesis of collagen proteins such as Collagen1 and α-SMA, thereby accelerating the pathological progression of silicotic fibrosis. This discovery elucidates how exosomal miRNAs can remodel the pulmonary microenvironment through precise regulation of the Ras signaling network, offering a novel potential target for the precise treatment of pneumoconiosis.

### 4.4 Crosstalk and network integration in miRNA-Mediated fibrosis

Rather than acting in isolation, the aforementioned miRNAs and their target pathways operate within a highly interconnected regulatory network. A converging mechanism across multiple studies is that different miRNAs ultimately drive or suppress the same core pathological events: EMT and FMT (Mehjabin et al., 2023). For instance, TGF-β1, serving as a master profibrotic hub, heavily crosstalks with PI3K/Akt, MAPK, and Wnt/β-catenin signaling cascades (Meng, Nikolic-Paterson, and Lan, 2016). miRNAs such as miR-34a-5p, miR-125a-3p, and miR-449a, although targeting different specific upstream receptors or kinases (e.g., Smad4, Fyn/STAT3, Bcl2), collectively converge on mitigating excessive ECM deposition by impeding this TGF-β1-driven network. Furthermore, the interplay between inflammatory-associated miRNAs (e.g., miR-146a, miR-125a-5p targeting NF-κB) and pro-fibrotic miRNAs illustrates a sequential and amplifying regulatory network where early immune imbalances seamlessly transition into irreversible fibrotic cascades. Recognizing these converging nodes is critical, as targeting a single miRNA might be bypassed by compensatory pathways, whereas intervening at convergent signaling hubs could offer more robust therapeutic efficacy.

Viewing this pathogenesis through a comprehensive systems biology perspective reveals that this intricate miRNA regulatory network is not spontaneous, but is actively driven by specific upstream regulators, predominantly oxidative stress and epigenetic modifications. Following the initial inhalation of occupational dust, the overproduction of ROS not only induces direct cellular damage but also acts as a potent upstream epigenetic trigger. This oxidative stress alters DNA methylation and histone acetylation landscapes, leading to the aberrant transcription of key miRNAs. Consequently, these upstream oxidative events initiate a highly integrated cascade: miRNAs regulating early inflammatory cytokines (such as IL-6 and TNF-α via the NF-κB pathway) directly crosstalk with the signaling cascades that govern late-stage extracellular matrix deposition (such as the TGF-β, PI3K/Akt, and MAPK pathways). This systems-level integration seamlessly connects the initial dust-induced inflammatory triggers to the ultimate profibrotic endpoints, forming a unified disease model.

## 5 Therapeutic applications, challenges, and future perspectives

miRNAs, as crucial molecules in the regulation of life processes, are not only involved in various important physiological regulatory mechanisms but also participate in the occurrence and progression of multiple diseases when abnormally expressed or dysfunctional. Pneumoconiosis is a progressive interstitial lung disease with a high mortality rate and limited clinical treatment options. Research over the past decades has revealed complex pathological mechanisms at the molecular and cellular levels in pneumoconiosis. Moreover, miRNA mimics have emerged as a research hotspot in the treatment of various diseases. Therefore, this review takes miRNAs as potential early biomarkers for pneumoconiosis as a starting point to analyze the roles of miRNAs in pneumoconiosis and their involvement in the process of pulmonary fibrosis associated with the disease.

Although miRNAs show great potential in the mechanistic study of pneumoconiosis, numerous challenges remain in translating laboratory findings into clinical applications:

1. Bottlenecks in Specificity and Standardization of Biomarkers: Current clinical research primarily focuses on differentially expressed miRNAs. However, due to the prolonged course of pneumoconiosis, significant heterogeneity exists in miRNA expression profiles across different dust types (e.g., silica, coal dust, asbestos) and various pathological stages (Tian et al., 2023). Furthermore, distinguishing pneumoconiosis-specific miRNAs from those associated with other interstitial lung diseases or chronic obstructive pulmonary disease remains a primary challenge for clinical diagnosis (Guiot et al., 2025). Additionally, the lack of standardized cross-laboratory detection protocols results in poor reproducibility of data across different studies. Particularly for exosome-derived miRNAs, the lack of consensus on extracellular vesicle (EV) isolation methods—ranging from traditional ultracentrifugation to size-exclusion chromatography and polymer-based precipitation—introduces significant variations in exosomal purity and miRNA yield, severely complicating biomarker validation (Llorens-Revull et al., 2023; Welsh et al., 2024).

To overcome these diagnostic bottlenecks, the future of clinical translation will inevitably shift from relying on single biomarkers to developing multi-marker diagnostic panels (Pirouzbakht, Hamzeh, and Soleimani Samarkhazan, 2025). While traditional clinical biomarkers for interstitial lung diseases—such as Krebs von den Lungen-6 (KL-6) and surfactant protein D (SP-D)—are well-established indicators of alveolar epithelial damage, they often lack sufficient specificity for early-stage occupational pneumoconiosis (Zuo et al., 2024; Tzang et al., 2025). Integrating specific miRNA signatures with these established protein biomarkers (e.g., an miRNA-KL-6/SP-D panel) could synergistically enhance both diagnostic sensitivity and specificity, providing a much more comprehensive evaluation of fibrotic progression than any single biomarker alone.

2. Challenges in Precision and Stability of miRNA Drug Delivery: When used as therapeutic targets, miRNA mimics or inhibitors are susceptible to degradation by nucleases *in vivo* (Setten, Rossi, and Han, 2019). For pneumoconiosis, achieving targeted pulmonary drug delivery, minimizing potential hepatorenal toxicity from systemic administration, and reducing off-target effects in non-target organs are crucial (Lam et al., 2015). While nebulized inhalation and nanocarrier delivery systems offer new directions, their delivery efficiency, long-term biocompatibility, and efficacy in large animal models require further validation (Yang et al., 2023). To address the stability and targeting challenges in these delivery systems, recent research has highlighted the transformative potential of EV-based platforms. Advances in exosome engineering technologies—such as surface modification and targeted cargo loading—have enabled EV-based biomarkers and exosome-mediated miRNA delivery systems to emerge as highly promising next-generation therapeutic carriers (Schwarz et al., 2025). Compared to traditional synthetic nanocarriers, these engineered exosomes demonstrate superior biocompatibility, low immunogenicity, and precise tissue-homing capabilities (Li et al., 2025; Xu et al., 2025).
3. Discrepancies Between Preclinical Models and the Human Physiological Environment: Current mechanistic studies largely rely on rodent models or *in vitro* cell cultures. However, animal models typically involve high-dose, short-term exposure, which fails to fully replicate the complex pathophysiological processes of human pneumoconiosis caused by decades of low-dose, repeated dust exposure (Sert and Yesil-Celiktas, 2025). This significantly limits the effective translation of laboratory findings into clinical treatments.
4. Integration of Single-Cell Transcriptomic Insights: Historically, most miRNA target analyses in pneumoconiosis have relied on bulk tissue sequencing, which masks critical cellular heterogeneity. Moving forward, single-cell transcriptomics presents a profound opportunity (Habermann et al., 2020). By mapping miRNA regulatory networks onto single-cell atlases, researchers can decipher how specific miRNAs govern the fate of emergent profibrotic cell clusters (e.g., transitional epithelial cells or specific macrophage subsets) (Strunz et al., 2020). Shifting from tissue-level to single-cell-level miRNA profiling will significantly enhance the biological interpretation of fibrotic mechanisms and facilitate the discovery of ultra-precise, cell-type-specific therapeutic targets.
5. Clinical Trial Status and Regulatory Hurdles: From a therapeutic perspective, the clinical translation of miRNA therapeutics for pneumoconiosis remains in its infancy. While several miRNA-targeted drugs for other conditions (e.g., oncology and viral hepatitis) have successfully entered clinical trials, investigations into miRNA interventions for occupational pulmonary fibrosis are still largely confined to preclinical validation (Winkle et al., 2021). The transition from bench to human trials is hindered by significant regulatory challenges. Regulatory agencies require standardized pharmacology and toxicology protocols specifically tailored for RNA-based therapies, definitive pharmacokinetic/pharmacodynamic (PK/PD) profiles, and clearly defined clinical endpoints for occupational fibrotic diseases (Christensen et al., 2025). Navigating these stringent regulatory pathways, alongside ensuring the long-term safety of systemic genetic modulation, is a critical prerequisite for advancing miRNA therapeutics to the bedside.

To address the challenge of diagnosing and treating pneumoconiosis, many scientists have shifted their focus to the role of genes in the development of the disease, discovering that miRNAs play a significant role in pneumoconiosis. Currently, differentially expressed miRNAs offer a novel approach for the early diagnosis of pneumoconiosis, providing a diagnostic technique with higher sensitivity, stronger specificity, and greater convenience for clinical practice. Although miRNAs have shown promise in the early treatment of pneumoconiosis, several issues remain insufficiently understood and require further in-depth research. These include how the target genes regulated by differentially expressed miRNAs are controlled within complex ceRNA networks, whether miRNAs exhibiting differential expression in pneumoconiosis show similar patterns in other diseases, and whether the onset of pneumoconiosis is solely related to specifically expressed miRNAs. Future studies should shift from single-miRNA paradigm toward network-based multi-omics approaches to decipher the holistic regulatory landscapes and identify core converging hubs for therapeutic interventions. In-depth research in this area not only offers a potential breakthrough for future clinical diagnosis and treatment of pneumoconiosis but also holds significant reference value for clinical drug development.
